# A Novel *TBK1* Variant (Lys694del) Presenting With Corticobasal Syndrome in a Family With FTD-ALS Spectrum Diseases: Case Report

**DOI:** 10.3389/fneur.2022.826676

**Published:** 2022-03-04

**Authors:** Kaitlin Seibert, Heather Smith, Allison Lapins, Peter Pytel, James A. Mastrianni

**Affiliations:** ^1^Department of Neurology, Center for Comprehensive Care and Research on Memory Disorders, University of Chicago, Chicago, IL, United States; ^2^Department of Pathology, University of Chicago, Chicago, IL, United States

**Keywords:** frontotemporal dementia, corticobasal syndrome, *TBK1*, motor neuron disease, dementia

## Abstract

Several variants of the TANK-Binding Kinase 1 (*TBK1*) gene have been associated with frontotemporal dementia - amyotrophic lateral sclerosis (FTD-ALS) spectrum diseases. Corticobasal syndrome (CBS) is characterized by asymmetric limb rigidity, dystonia or myoclonus, in association with speech or limb apraxia, cortical sensory deficit, and/or alien limb. It can result from a variety of underlying pathologies and although typically sporadic, it has been occasionally associated with *MAPT* and *GRN* variants. We describe here the proband of a family with multiple occurrences of FTD-ALS spectrum disease who developed an isolated right-sided primary asymmetric akinetic-rigid syndrome and subsequent speech and cognitive dysfunction associated with contralateral anterior temporal lobe atrophy on MRI and corresponding hypometabolism by FDG-PET. Genetic testing revealed a novel Lys694del variant of the *TBK1* gene and Type A TDP-43 pathology in a predominantly frontotemporal distribution contralateral to the affected side. To our knowledge this is the first report of CBS as the initial expression of a *TBK1* variant. This case emphasizes the importance of considering *TBK1* genetic screening in patients with CBS, as this may be an underrepresented population on the spectrum of genetic FTD-ALS.

## Introduction

Corticobasal syndrome (CBS) is a clinical syndrome characterized by asymmetric limb rigidity, dystonia or myoclonus, in association with speech or limb apraxia, cortical sensory deficit, and/or alien limb. Although most commonly considered a tauopathy and associated with the underlying pathology of corticobasal degeneration, a subtype of frontotemporal dementia, a small portion of cases are attributable to other pathologies (1). We present a patient with CBS and corresponding TDP-43 pathology, the primary pathology most closely linked to ALS (2). Not only is the clinicopathological correlation of CBS and TDP-43 unusual, but our case also carried a novel mutation in the TANK binding kinase 1 gene (*TBK1*) that is central to the development of ALS spectrum diseases (3). *TBK1* plays a role in autophagy and variants of the gene are associated with impaired autophagy that leads to accumulation of TDP-43 that commonly results in an FTD-ALS syndrome; however, our patient presentated with CBS and lacked EMG findings of ALS (1, 4). This case serves to expand the clinical phenotype of FTD-ALS spectrum diseases.

## Case Report

A 71-year-old right-handed man with hypertension, hyperlipidemia and glaucoma developed progressive right arm stiffness and dysarthria over the course of 1 year. He presented to the emergency department for an episode of dysarthria and elevated blood pressure, although a brain MRI lacked evidence of a stroke. Three months later, he was noted to have mild guttural dysarthria, significantly increased tone in his right arm that he held in flexion, and difficulty extending and abducting his fingers on the right hand, although strength was full throughout the arm. Reflexes were slightly brisk in the right arm, with upgoing plantar reflexes bilaterally. He walked with a slightly stooped posture and reduced right arm swing and he exhibited mild postural instability, although stride length and gait speed were normal. Cursory assessment of orientation, naming, sensory and cerebellar function was intact. Review of a prior brain and spinal MRI revealed left, greater than right, mild subcortical white matter hyperintensities and left, greater than the right, mild temporal lobe atrophy in addition to a moderate degree of spinal stenosis with severe bilateral C5-C7 foraminal stenosis, right worse than left. An electromyogram (EMG) of the right upper extremity and cervical paraspinal muscles were, however, normal. He underwent intensive physical, occupational and speech therapy with some improvement in daily function.

**Figure 1:**
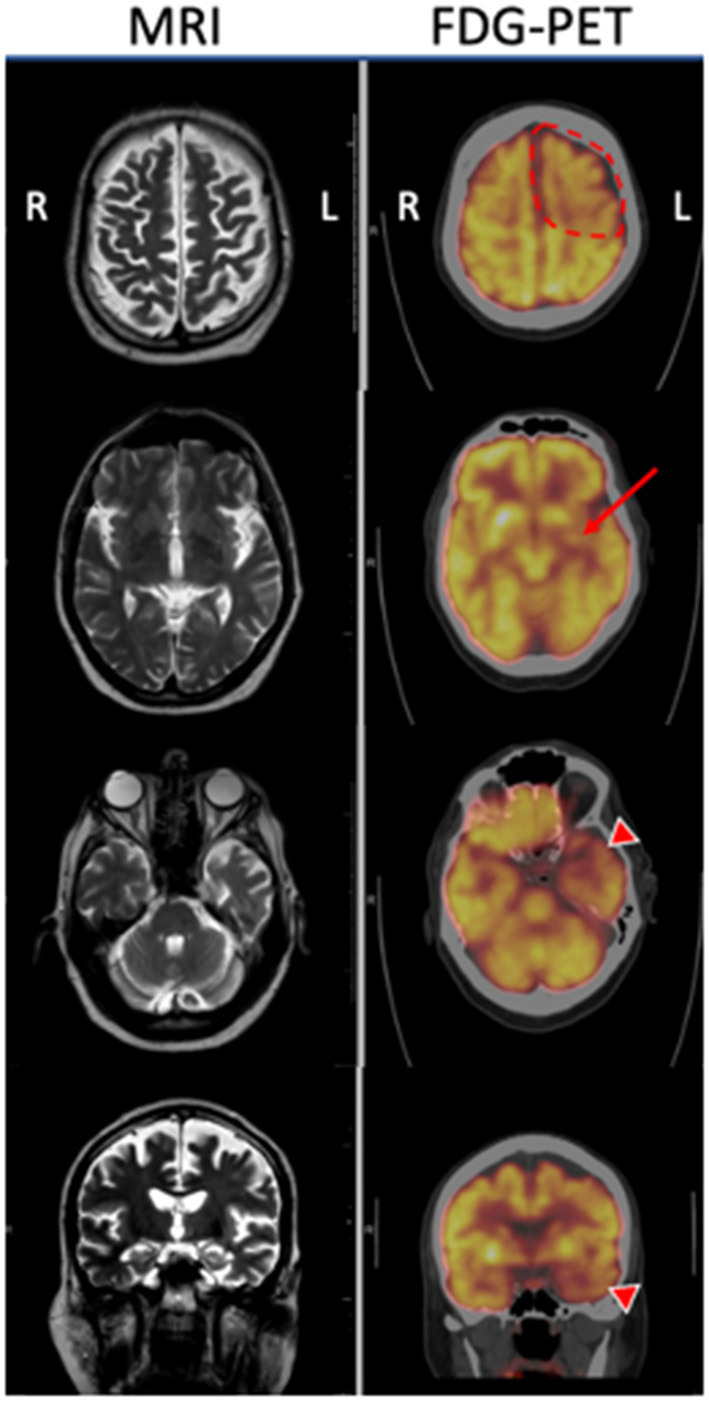
MRI T2 sequences and corresponding FDG-PET imaging of the proband at three axial levels and a coronal image through the hippocampi. MRI demonstrates generalized cortical atrophy of the left hemisphere, especially evident within the left anterior temporal lobe. FDG-PET reveals hypometabolism of left frontal lobe (dotted line) that was more prominent than the right, in addition to the left putamen (arrow), and left temporal lobe (arrowheads) compared with the corresponding regions on the right.

**Figure 2:**
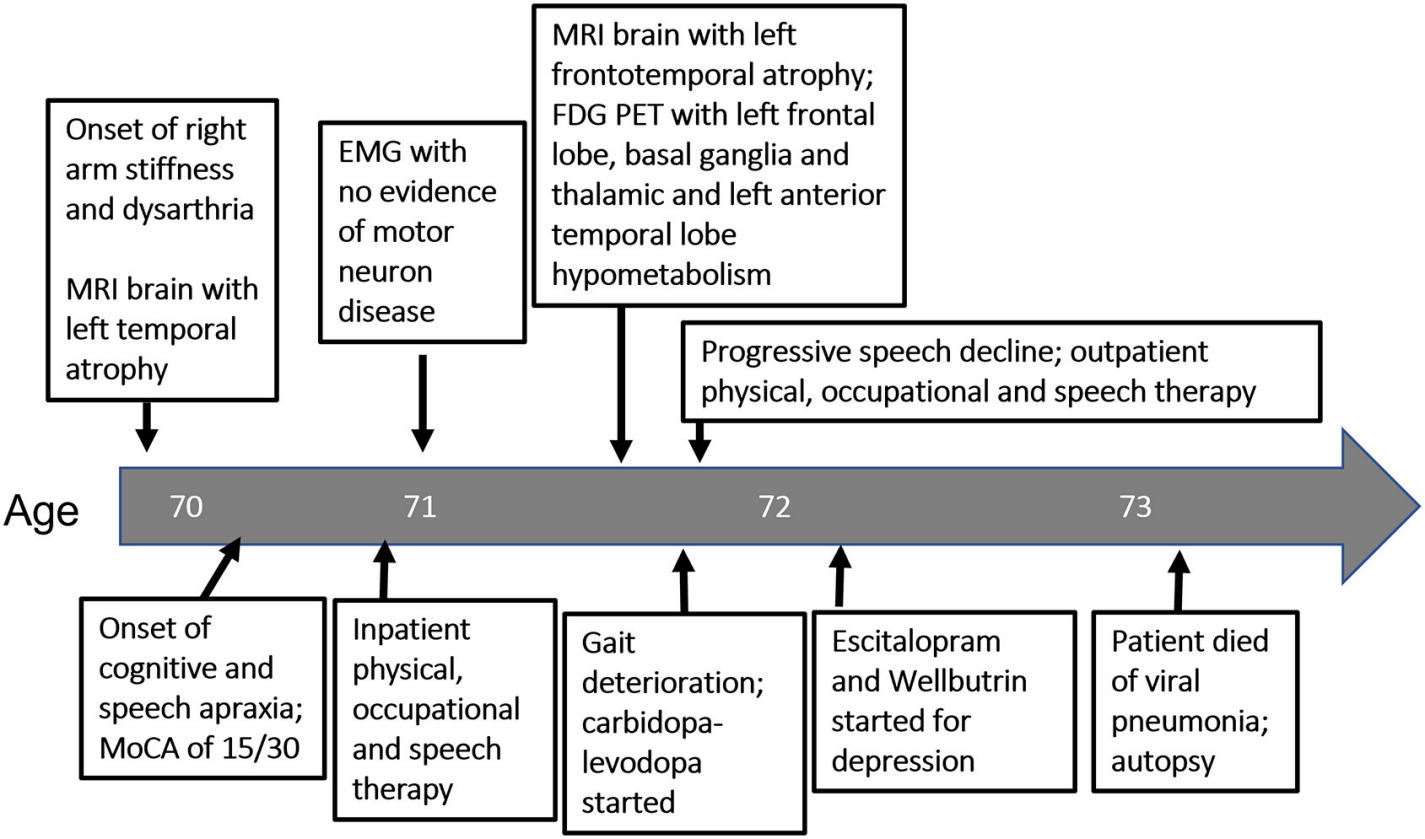
Timeline of diagnostic testing and intervention from symptom onset (left) to patient death (right) as it relates to patient's age.

Three months later he developed worsening dysarthria with right-sided facial droop and dysphagia. The rigidity of the right upper extremity was more pronounced and now extended into his right leg. At this time he scored 15 on the Montreal Cognitive Assessment (MoCA), with deficits primarily involving executive function, attention, visuospatial ability and delayed recall that did not improve with cueing. A formal speech evaluation by a neurological speech therapist revealed a strained hypernasal and breathy vocal quality with a slow, effortful and segmented speech pattern. He exhibited frequent misarticulations, imprecision with consonants and he produced only one syllable per breath, with elements of both spastic and flaccid dysarthria. He displayed ideomotor apraxia in his right arm. A three-limb, cervical and thoracic paraspinal muscle and tongue EMG performed a year after the initial one revealed only focal denervation in the right flexor carpi radialis and no evidence of tongue denervation as might be expected in ALS. A comprehensive laboratory assessment for a primary metabolic, infectious, or inflammatory condition was negative. Follow-up brain MRI revealed mild progression of atrophy, predominantly within the left motor and pre-motor cortex and left temporal lobe (Figure 1). A brain fluorodeoxyglucose-positron emission tomography (FDG-PET) scan demonstrated left hemisphere hypometabolism primarily within frontal lobe, basal ganglia, and thalamus, supportive features of CBS (Figure 1). Hypometabolism of the left anterior temporal lobe was also noted. CSF studies were negative for markers of infection, inflammation, or autoimmunity due to paraneoplastic or non-paraneoplastic causes. CSF biomarkers for Alzheimer's Disease were performed, with equivocal results. Although the Aβ/tau index of 0.54 (Aβ42 = 375.9 ng/mL, total tau = 389.4 ng/mL) fell below the threshold of ≤ 0.8 that supports Alzheimer's Disease, the phosphorylated tau level of 38.55 pg/mL was well below the lower threshold of 68 pg/mL that would support Alzheimer's Disease. He tolerated carbidopa-levodopa, which was gradually increased to 50/200 mg three times a day over a few months with some improvement in gait instability. Baclofen 10 mg daily was ineffective and was stopped after several weeks. The mainstays of treatment were physical, occupational and speech therapy. Rehabilitation improved his swallowing technique to reduce the risk of aspiration, provided targeted exercises to preserve his mobility, and supplied him with compensatory strategies for cognition. He was also provided specialized devices to communicate with his family. He used a communication board to spell “depressed” at a subsequent visit, at which time escitalopram 10 mg and buproprion XL 150 mg per day were trialed without improvement per family report. After 3 years of disease progression, the patient died at home from viral pneumonia. A timeline of his clinical course can be seen in Figure 2.

**Figure 3:**
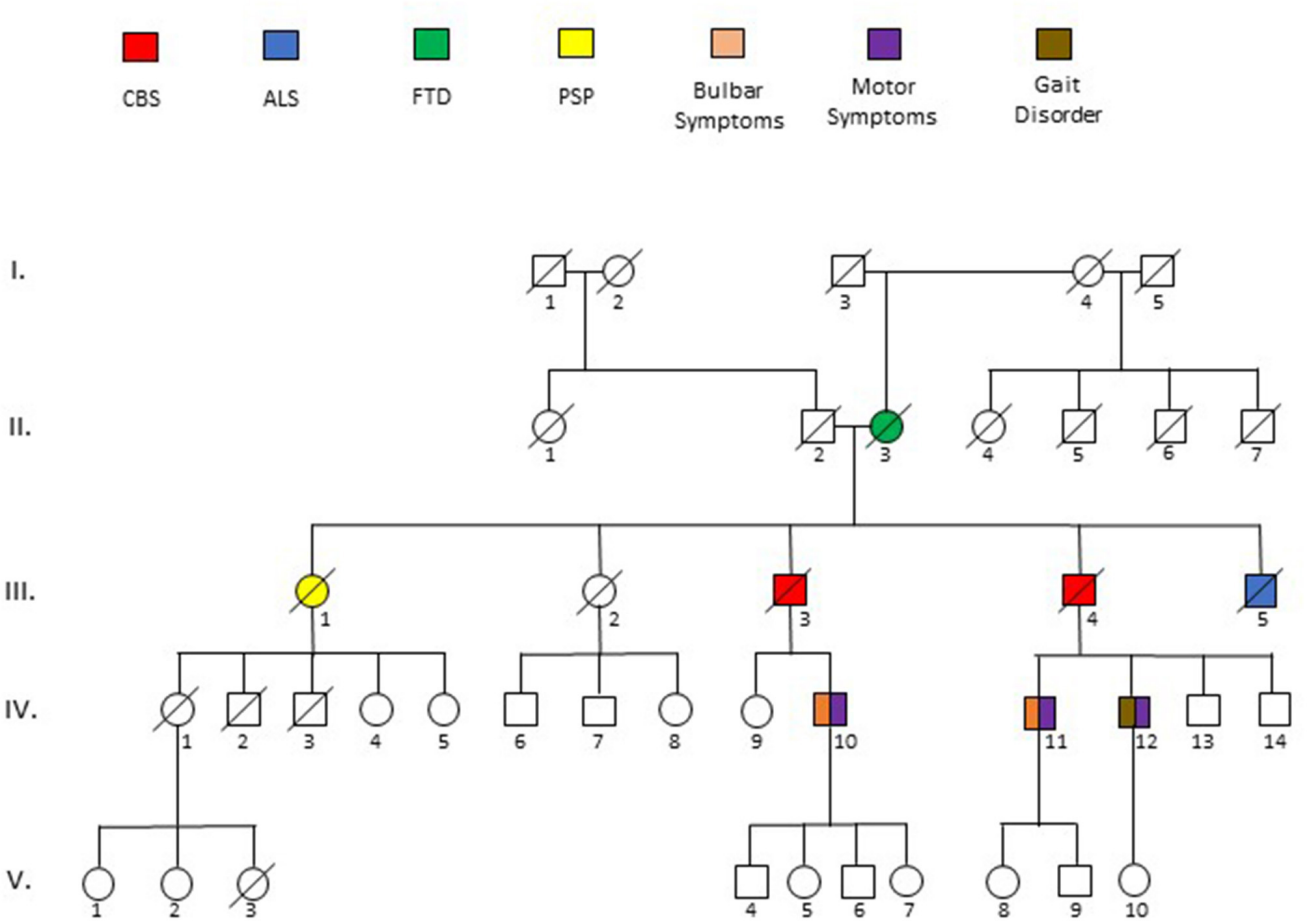
Pedigree of five generations (I-V) surrounding the sentinel patient (III.3). Phenotypes are described by key above pedigree. Deceased patients are indicated by a diagonal slash. Ages at death as follows: II.3 = 73, III.1 = 55, III.3 = 72, III.4 = 62, III.5 = 63. Specific details of the description of disease phenotypes of affected individuals is described in the text.

The patient had an extensive family history of neurological disorders (Figure 3). His mother (II.3) died at 76 years after a 3-year course of progressive personality change with erratic behavior and paranoia that eventually required admission to a psychiatric facility. His father (II.2) and paternal siblings were unaffected. Each of his four siblings died before the age of 65; three of which were attributed to a neurological disease. One brother (III.4) died after 5 years of progressive dysarthria, dysphagia, right arm stiffness and inability to ambulate, although he was placed in a nursing home at the start of his disease and was never formally diagnosed. Another brother (III.5) died in his 50s of ALS that was established by an EMG performed for progressive leg weakness and atrophy, and a sister (III.1) died with a diagnosis of progressive supranuclear palsy (PSP) at age 55, the details of which are unknown. Another sister (III.2) passed away at age 61 with diagnosis of systemic Lupus Erythematosus (SLE). A son (IV.11) of the brother (III.4) who died with a similar phenotype as the proband was labeled with Huntington's disease following development of coordination difficulty, dysphagia, and myoclonus at 38 years of age. Another son (IV.12) of that same brother developed unilateral arm stiffness/pain and gait dysfunction in his 50s, very similar to the proband. None of these conditions were pathologically or genetically confirmed.

Based on the strong family history, genetic testing of a panel of FTD-ALS and CBS associated genes was performed: *C9orf72, MAPT, CHCHD10, DCTN1, FUS, TARDBP, UBQLN2, VCP, CHMP2B, HNRNPA2B1, MATR3, SIGMAR1, SQSTM1, ALS2, APP, KIF5A, OPTN, PFN1, PRNP, PSEN1, PSEN2, SNCA, SOD1, SPG11, TFG, VAPB*, and *GRN* were normal, but a previously unreported heterozygous c.2080_2082del variant within the *TBK1* gene that results in a Lys694del change in the protein was detected. Another unreported variant was detected in the *SETX* gene c.4433 C>A (p.Ala1478Glu).

The differential diagnosis for our patient was broad, as he initially demonstrated both extrapyramidal and pyramidal signs and symptoms. Motor neuron disease was a strong consideration. Initially his dysphagia and speech trouble seemed to be a bulbar problem, but later was realized to be an orolingual apraxia. However, the presence of sensory symptoms and the relative lack of denervation on EMG despite significant speech and unilateral motor symptoms argued against this as an etiology. Rather, the insidious and gradually progressive akinetic rigid syndrome with cortical sensory features, speech and language impairment, asymmetric dystonia and frontal-executive dysfunction without sustained response to levodopa treatment meets current clinical criteria for corticobasal syndrome (1). FDG-PET was critical in confirming the diagnosis, as it reflected dysfunction in an asymmetric pattern thatinvolved the thalamus, basal ganglia and cortex, most representative of his corticobasal syndrome-like presentation. Despite the lack of a genetic variant of *APP, PSEN1 or PSEN2*, and the low p-tau in CSF, a variant of Alzheimer's disease with motor features remained a possibility, until autopsy.

**Figure 4:**
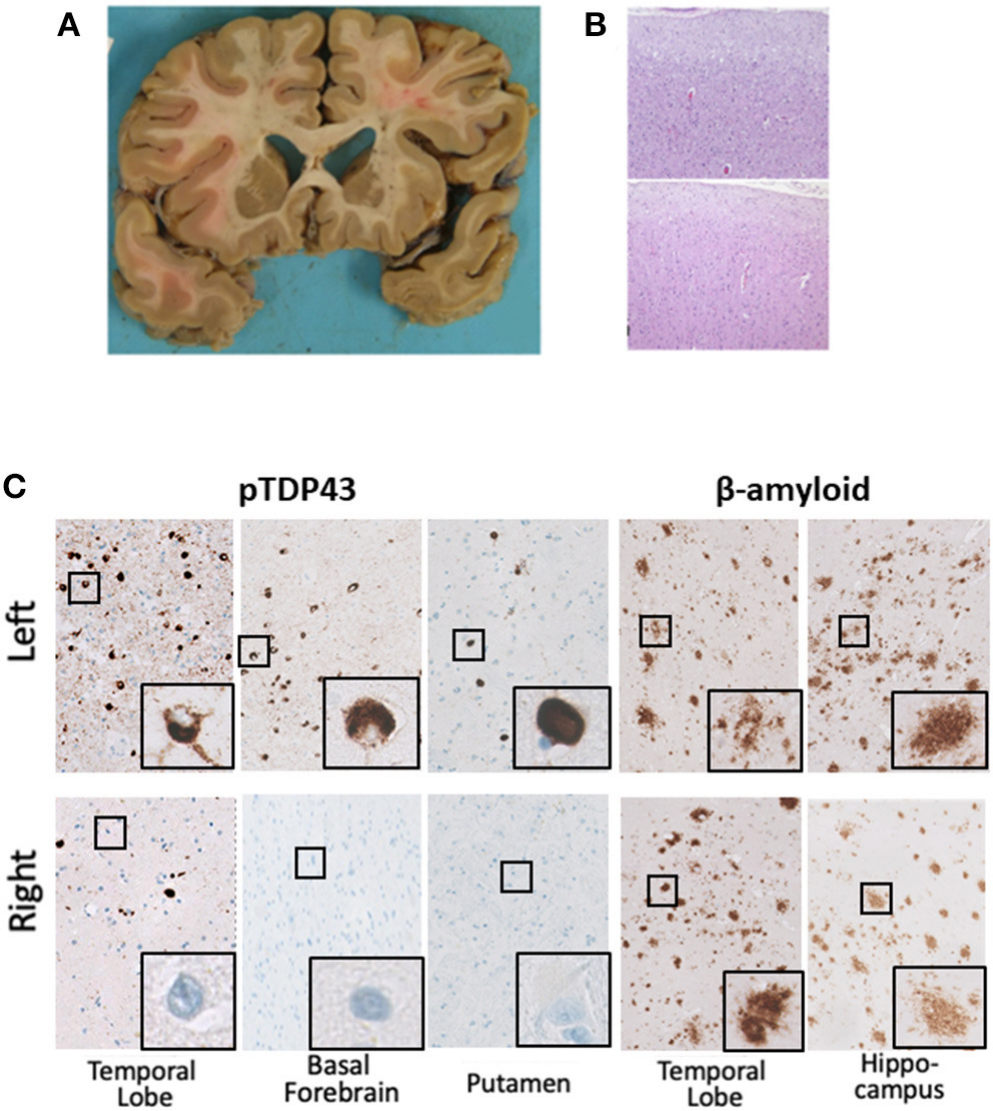
**(A)** Coronal section of brain from the proband. Gross examination revealed atrophic changes in the frontal, temporal, and insular cortices, in addition to basal ganglia, all of which were more pronounced on the left compared with the right. **(B)** H&E examination of the lateral left (top) and right (bottom) frontal cortex shows mild spongiform changes that were especially prominent in the superficial layers, a common and non-specific finding in patients with neurodegenerative disease. **(C)** Immunohistochemical stains for pTDP-43 reveal asymmetrically distributed perinuclear pTDP-43 inclusions in the left temporal lobe, basal forebrain, and basal ganglia. β-amyloid plaques were evenly distributed in the left and right temporal cortices and left and right hippocampi.

A post-mortem study was performed on the brain and spinal cord to determine the primary underlying pathologic process (Figure 4; Table 1). Gross examination of the brain revealed mild cortical atrophy most prominent throughout the left hemisphere. The left insular and anterior temporal cortex were most affected, followed by left frontal and parietal cortices (Figure 4A). Histological findings on H&E staining revealed non-specific spongiotic changes greater in the left than the right superficial cortices (Figure 4B). Immunohistochemical stains for TDP-43, pTDP-43, alpha synuclein, tau (4R and 3R), and β-amyloid were performed bilaterally in the hippocampus, temporal cortex, frontal cortex, parietal cortex, occipital cortex, and basal ganglia (Table 1). Staining for pTDP-43 confirmed the presence of prominent inclusions that followed the same general distribution as the asymmetric atrophy (Figure 4C). TDP-43 was predominantly present as perinuclear neuronal cytoplasmic inclusions with relatively few dystrophic neurites. These were most prominent within the superficial layers of the cortex. They were also present within neurons of the dentate gyrus as well as pyramidal neurons within the left hippocampal formation, in addition to the left lentiform nucleus. The overall pattern of pathology best matches that described as Type A TDP-43 pathology (2).

**Table 1:** Relative levels of histopathologic changes in the brain of a patient with CBS and a Lys694del variant of the TBK1-gene.

**Brain region**	**Hematoxylin and eosin**	**TDP-43/pTDP-43**	**alpha-synuclein**	**Tau (4R)**	**Tau (3R)**	**β-amyloid**
Midbrain	No Lewy bodies or decrease in pigmented neurons	ND	–	ND	ND	+
Putamen/globus pallidus	Minimal changes	+++ (L), + (R)	ND	ND	ND	ND
L. Hippocampus	Few Hirano bodies, rare granulovacuolar degeneration, no hippocampal sclerosis	+++	–	++	+	++
R. Hippocampus	Rare granulovacuolar degeneration, no hippocampal sclerosis	+	–	++	+	++
L. temporal cortex	Superficial vacuolization of cells	+++	–	Sparse, superficial tangles	–	+++
R. temporal cortex	Minimal changes	+	–	+	–	+++
L. frontal cortex	Vacuolar changes, especially in the superficial cortex	+++	ND	+	–	+++
R. frontal cortex	Mild vacuolization in the superficial cortex	+	ND	–	–	+++
R. parietal cortex	Minimal changes	ND	ND	–	–	+++
R. medial occipital	Minimal changes	ND	ND	ND	–	++
Cerebellum	Normal	ND	ND	ND	ND	Amyloid angiopathy only

*+, mild; ++, moderate; +++, severe; –, negative; ND, not done*.

Interestingly, TDP-43 pathology was superimposed on a background of β-amyloid (Aβ) and tau accumulation. Cortical Aβ deposits took the form of diffuse plaques, neuritic plaques, focal subpial deposits, and amyloid angiopathy, as summarized in Table 1. However, in contrast to the predominance of TDP-43 pathology within the left hemisphere, Aβ pathology was symmetrically distributed. Neurofibrillary tangles were largely restricted to the pyramidal layer of the hippocampus as well as the entorhinal cortex, with only focal sparse tangles in the lateral temporal cortex and frontal cortex (see Table 1). Four-repeat (4R) tau predominated, with only minimal staining for three-repeat (3R) tau. Overall, the Alzheimer-related neuropathologic changes fit A3B2C2 classification (5, 6).

## Discussion

We describe the clinical, pathologic, and genetic features of a patient who presented with CBS and carried a novel mutation of the TANK-binding kinase 1 (*TBK1*) gene. This case not only expands the list of known disease-associated *TBK1* variants, but it extends the phenotypic spectrum with which it is associated.

CBS is characterized by the development of progressive asymmetric parkinsonism, cortical deficits such as alien limb phenomenon or limb apraxia, and cognitive dysfunction, often associated with language or speech dysfunction. Neuroimaging that displays asymmetric frontoparietal atrophy on MRI or hypometabolism within the frontoparietal cortex, basal ganglia and thalamus of the contralateral hemisphere on FDG-PET supports the diagnosis (7, 8). Our patient's disease was heralded by the development of an asymmetric akinetic-rigid syndrome involving the right arm that eventually spread to the right leg, involved speech and language, in addition to apraxia and mild cognitive decline. Additionally, our patient had left hemiatrophy on MRI and left hypometabolism on FDG-PET, which correlated well with his right-sided clinical syndrome. By all clinical parameters, this patient met the clinical diagnosis of CBS.

Because CBS can occur in association with several underlying diseases, including Alzheimer's disease (AD), corticobasal degeneration (CBD), progressive supranuclear palsy (PSP), or primary progressive aphasia (PPA), among others (8, 9), some have suggested the FDG-PET can be helpful in predicting the underlying disease. Pardini et al. (8) found that precentral gyrus hypometabolism is a consistent feature of underlying CBD, whereas a more posterior pattern of asymmetric hypometabolism involving the lateral temporal and parietal lobes along with posterior cingulate gyrus are more commonly associated with AD, and a more anterior hypometabolic pattern involving medial frontal lobes and anterior cingulate gyrus suggests underlying PSP. Others have found that hypometabolism in CBS can also involve the lateral premotor cortex, supplementary motor area, prefrontal lobes, superior parietal lobes, striatum and thalamus (10). Our patient's FDG-PET revealed asymmetric hypometabolism of the left precentral gyrus and surrounding frontoparietal cortex, in addition to the left basal ganglia and thalamus. Additionally, hypometabolism within the left anterior temporal lobe was present, a feature often associated with the semantic variant of Primary Progressive Aphasia (PPAsv) and which was not common to any of the described pathologies in Pardini's series (8). Although a single case, it is interesting to consider that the anterior temporal pole involvement, with or without language dysfunction, might be a potential biomarker of a *TBK1*-based ALS-FTD spectrum disorder.

The histopathological findings of this patient suggest TDP-43 pathology is the cause of his clinical syndrome. The marked asymmetry of TDP-43 deposition lateralized to the left premotor and motor cortex strongly argues this pathology to be responsible for his right-sided presentation of CBS. Although Aβ plaques and NFTs were also present, these were more generalized in their distribution. In fact, the presence of 4R tau and Aβ plaques are not uncommon in TDP-43 FTD-ALS spectrum disorders and seem to be an age-related phenomenon, correlating more with disease severity rather than clinical presentation (11). Although 4R-tau is more commonly linked to CBD, we saw no evidence of CBD-like tau-positive glial inclusions or ballooned neurons.

The previously unreported Lys694del variant of the *TBK1* gene identified in this patient, in the absence of alterations in *C9orf72, MAPT, CHCHD10, DCTN1, FUS, TARDBP, UBQLN2, VCP, CHMP2B, HNRNPA2B1, MATR3, SIGMAR1, SQSTM1, ALS2, APP, KIF5A, OPTN, PFN1, PRNP, PSEN1, PSEN2, SNCA, SOD1, SPG11, TFG, VAPB*, and *GRN*, and the extensive family history, supports a causal role of the mutation and an autosomal-dominant pattern of inheritance. Affected family members were all in their 50s or early 60s and they developed a clinical syndrome that fit within FTD-ALS spectrum or, as in the case of his brother (III.4) and his brother's son (IV.12), a remarkably similar presentation to that of the proband. Although his mother was reportedly diagnosed with AD, her behavioral features suggest she likely had FTD. The patient's maternal grandmother (I.4) and her half siblings (II.3-6) were unaffected and, although his grandfather (I.3) was estranged from the family, based on the lack of disease in patients I.1, I.2, and I.4, the patient's mother may have been the first to harbor or express the variant *TBK1* gene. We considered the variant in *SETX* unlikely to have contributed to our patient's clinical syndrome, as this gene is typically implicated in juvenile-onset ALS and autosomal recessive cerebellar ataxias; however, we cannot rule out a role, as an oligogenic basis of FTD-ALS spectrum disorders has been proposed by some (12, 13).

Currently, over 70 variants of the *TBK1* gene have been found in association with ALS and FTD (3), yet the full scope of phenotypic variance has not yet been realized (14). Caroppo et al. (15) described two carriers of c.1446T.G (p.Tyr482X) and c.1963C.T (p.Gln655X) mutations who presented with FTD-PPAsv and ALS, and two with variants of c.467_468delCA(p.Thr156ArgfsX6) and c.1960-2A>G (p.Leu654LysfsX18) who developed the progressive non-fluent aphasia variant of FTD (i.e., FTD-PPA-agrammatic) in addition to CBS and ALS, respectively. Interestingly, those patients displayed anterior temporal and opercular atrophy on MRI, as in our case. More recently, a Glu703X variant of *TBK1* was reported in a patient who developed PPA-agrammatic that progressed to CBS, with autopsy-confirmed Type A TDP-43 histopathology (16).

Of the 11 reported cases with histopathologic confirmation of disease, abnormal deposition of TDP-43 has been uniformly present, with eight displaying Type B pathology (12, 17–20) and three with Type A (13, 16, 21). Two cases demonstrated 4R-tau; one with hyperphosphorylated tau with features of argyrophilic grains with neurofibrillary tangles in the hippocampus accompanied by grains in the hippocampal CA1 subregion and the subiculum, in addition to small numbers of bushy astrocytes in cortical regions (16) and one with 4R tau in the amygdala, entorhinal and transentorhinal cortex, CA1 sector of the hippocampus, and cingulum (19). Our case suggests that a pure CBS phenotype should also be included within the heterogeneous pool of *TBK1* variants.

Although the Lys694del variant could not be causally segregated with disease in this family, as a result of the early deaths of affected family members, the causal nature of this variant is supported by its similarity to previously reported cases, including CBS phenotypes with anterior temporal lobe hypometabolism and similar pathology, and the patient's extensive family history of FTD-ALS spectrum disorders. The single amino acid deletion of the Lys at residue 694 of TBK1 is likely to be pathogenic, as it lies within the coiled-coil domain 2 (CCD2) that extends from residues 658–713, a critical region for autophagy-related protein binding and function (22, 23). Several missense and deletion variants within this domain have been associated with an FTD-ALS phenotype. For example, a p.690-713 deletion associated with ALS and FTD was reported to result in a loss of function variant that prevented the binding of optineurin (OPTN) to TBK1 protein (23). A similar haploinsufficiency effect was proposed for the Glu703X mutation (16). In one study, alterations within the CCD2 domain were found to, more likely than other domains, cause ALS-FTD or FTD rather than pure ALS (23). The primary mechanism by which mutations induce disease is thought to be via impaired autophagy, as a result of haploinsufficiency of *TBK1*, among others (22, 23). A potential mechanism relates to impaired localization of TBK1. The CCD2 domain of TBK1 binds to adaptor proteins NAP1, TANK, and Sintbad in a mutually exclusive fashion. Adaptor proteins are thought to localize TBK1 into particular cellular compartments, thereby determining the pathway that TBK1 will participate (23). Decreased expression and impaired localization of TBK1 protein may lead to impaired kinase activity, impaired selective autophagy, and resultant protein aggregation, thereby facilitating neurodegeneration (24, 25). While the mechanism underlying specific deposition of TDP-43 in patients with *TBK1* variants is incompletely understood, failure of p62-mediated autophagy and upregulation of NF-KB inflammatory pathways resulting from *TBK1* variants may play a role in the deposition of TDP-43 and resultant FTD-ALS pathogenesis (26).

The strengths of our case include access to critical diagnostic tests such as EMG, FDG-PET and genetic testing. Additionally, our clinical diagnosis was confirmed by post-mortem evaluation, which showed markedly asymmetric TDP-43 pathology in addition to diffuse Alzheimer's pathology. A limitation of our case is that all of the patient's symptomatic relatives died before him and did not have autopsies, so co-segregation of the *TBK1* variant with diseasepathogenicity could not be confirmed. Additionally, this patient's perspective on his disease and neuropsychological testing were not obtained prior to his passing.

In summary, we describe the proband of a family affected by FTD-ALS spectrum diseases who presented with CBS in association with a novel mutation of the *TBK1* gene and TDP-43 Type A pathology. This case provides additional support for the role of *TBK1* in the development of FTD spectrum disease and extends the range of disease to include CBS.

## Data Availability Statement

The original contributions presented in the study are included in the article/supplementary material, further inquiries can be directed to the corresponding author/s.

## Author Contributions

KS drafted the manuscript with assistance from AL. HS and PP assisted with pathology figures and content. JM wrote and edited the manuscript with assistance from KS and AL. All authors contributed to the article and approved the submitted version.

## Conflict of Interest

The authors declare that the research was conducted in the absence of any commercial or financial relationships that could be construed as a potential conflict of interest.

## Publisher's Note

All claims expressed in this article are solely those of the authors and do not necessarily represent those of their affiliated organizations, or those of the publisher, the editors and the reviewers. Any product that may be evaluated in this article, or claim that may be made by its manufacturer, is not guaranteed or endorsed by the publisher.
